# Interleukin 15 Primes Natural Killer Cells to Kill via NKG2D and cPLA2 and This Pathway Is Active in Psoriatic Arthritis

**DOI:** 10.1371/journal.pone.0076292

**Published:** 2013-09-25

**Authors:** Fangming Tang, Benjamin Sally, Cezary Ciszewski, Valerie Abadie, Shane A. Curran, Veronika Groh, Oliver FitzGerald, Robert J. Winchester, Bana Jabri

**Affiliations:** 1 Department of Medicine, University of Chicago, Chicago, Illinois, United States of America; 2 Department of Microbiology and Immunology, Columbia University, New York, New York, United States of America; 3 Department of Medicine, Columbia University, New York, New York, United States of America; 4 Clinical Research Division, Fred Hutchinson Cancer Research Center, Seattle, Washington, United States of America; 5 Department of Rheumatology, St. Vincent’s University Hospital and Conway Institute, University College Dublin, Dublin, Ireland; INSERM- CNRS- Univ. Méditerranée, France

## Abstract

NK cells are large granular lymphocytes that form a critical component of the innate immune system, whose functions include the killing of cells expressing stress-induced molecules. It is increasingly accepted that despite being considered prototypical effector cells, NK cells require signals to reach their full cytotoxic potential. We previously showed that IL-15 is capable of arming CD8 effector T cells to kill independently of their TCR via NKG2D in a cPLA2-dependent process. As NK cells also express NKG2D, we wanted to investigate whether this pathway functioned in an analogous manner and if resting NK cells could be primed to the effector phase by IL-15. Furthermore, to establish relevance to human disease we studied a possible role for this pathway in the pathogenesis of psoriatic arthritis, since there are aspects of this disease that suggest a potential effector role for the innate immune system. We found that PsA patients had upregulated IL-15 and MIC in their affected synovial tissues, and that this unique inflammatory environment enabled NK cell activation and killing via NKG2D and cPLA2. Moreover, we were able to reproduce the phenotype of joint NK cells from blood NK cells by incubating them with IL-15. Altogether, these findings suggest a destructive role for NK cells when activated by environmental stress signals during the pathogenesis of PsA and demonstrate that IL-15 is capable of priming resting NK cells in tissues to the effector phase.

## Introduction

One of the major functions of the innate immune system is to identify and respond to stressed cells. A key molecule in the stress pathway is major histocompatibility complex class I chain-related A (MICA), which is upregulated on cell surfaces in response to challenges to cellular homeostasis [1]. MICA is recognized by an activating C-type lectin domain-containing NK receptor called natural killer group 2 member D (NKG2D) that is expressed on both natural killer (NK) cells and cytotoxic T cells, promoting the killing of stressed targets [2]. We have shown previously that this process is dependent on cytosolic phospholipase A2 (cPLA2) in effector CD8 T cells [3], though this has never been demonstrated in NK cells. NKG2D has been implicated in the pathogenesis of numerous human autoimmune disorders, including but not limited to celiac disease [3-5], rheumatoid arthritis [6], systemic lupus erythematosus [7], and Crohn’s disease [8]. However, despite its classification as an NK receptor, NKG2D has primarily been studied in autoimmunity in the context of T cells [9].

NK cells are large granular lymphocytes that form a critical component of the innate immune system due to their cytolytic capabilities and their capacity to release various cytokines. They are primarily thought to play a role in immune surveillance, wherein they lyse cells that have downregulated HLA ligands in response to intracellular infection [10]. Increasingly however, a potential role for NK cells is being investigated in the pathogenesis of various immune disorders, as their presence at a high frequency has been noted in sites of inflammation [11].

Psoriatic arthritis (PsA) is a unique inflammatory arthritis associated with the chronic skin disorder psoriasis, estimated to afflict between 0.3 and 1.0% of the population [12]. Efforts to decipher the immune mechanisms underlying PsA have led to several paradoxes. Genetic susceptibility is marked and strongly associated with certain class I allotypes, suggesting the operation of an adaptive immune response [13]. This is further supported by the finding of increased CD8 T cells in the joint, and notable clonal expansions within the CD8 compartment [14,15]. However, the class I MHC susceptibility includes several disparate and structurally unrelated HLA allotypes, and structural analysis of the expanded T cell clones during active synovitis, therapy-induced remission, and potentially their subsequent recrudescence has failed to identify persistent structurally related driver clones in these phases of the disease [15]. This is inconsistent with the usual concept of a unitary T cell clonally driven adaptive autoimmune process. Also difficult to reconcile are the observations that severe PsA may develop *de novo* in the setting of advanced acquired immune deficiency syndrome, when the immune system has limited capacity to initiate an adaptive response [16]. Taken together, these findings underscore the confounding aspects of the adaptive immune response in PsA and suggest that other components of the immune system, most likely those of the innate branch, may have an active effector role during disease pathogenesis [16,17].

Previous studies have shown that synovial NK cells from patients with chronic forms of arthritis, including rheumatoid arthritis, non-specific polyarthritis, PsA, and ankylosing spondylitis (AS) have an activated phenotype as evidenced by an increase in CD69 expression and lower levels of killer-cell immunoglobulin-like receptors (KIRs) [18](FitzGerald unpublished data). In this study, we investigate the role of cPLA2 in NKG2D-mediated cytolysis in NK cells and the possibility that NKG2D may be aberrantly activated in synovial NK cells by environmental changes in the joint tissue in the setting of PsA. We selected AS as a comparison disease because it also has associates with the class I allotype HLA-B*27 while exhibiting other distinctive features in its immunopathogenesis that distinguish it from PsA [19].

## Materials and Methods

### Ethics statement

All human subjects gave written informed consent and all protocols were approved by the Columbia University Medical Center institutional review board.

### Human subjects

All hematopoietic cells from blood and joint fluid fractions were from PsA and AS patients. For immunohistochemical studies, six adult patients with PsA or AS and four age-matched control individuals undergoing biopsies for functional disorders of non-psoriatic arthritis were studied. In addition, NK cells were isolated from the joint fluid of individuals with PsA.

Paired blood and joint fluid samples were obtained from six patients with active psoriatic arthritis undergoing therapeutic knee aspirations. All patients had plaque psoriasis and their disease duration ranged from 2.0 to 10.5 years.

### Immunohistochemistry

Immunohistochemical staining for MIC and IL-15 was performed on 4 µM 10% formalin-fixed paraffin sections using a double staining blocking kit (DAKO). Monoclonal mouse anti-MIC (1:200 dilution) and anti-IL-15 (1:500 dilution) antibodies were used, both from Abcam.

### Fresh NK isolation, NK clone generation, and cell culture

Primary human NK cells were isolated from peripheral blood from healthy donors by depletion of non-NK cells (negative selection kit, Miltenyi Biotec). In brief, peripheral blood lymphocytes were isolated after Ficoll density gradient centrifugation (Amersham Pharmacia Biotech, Piscataway, NJ). Non-NK cells were magnetically labeled indirectly with a cocktail of biotin-conjugated antibodies against lineage-specific antigens and a cocktail of microbeads. Unlabeled cells that passed through the MACS column were collected. Purity of NK cell isolation (>95%) was confirmed by flow cytometry and cells were cultured with IL-2 (500 unit/mL) in growth medium.

Enriched CD16+ CD56+ NK cells were cloned as previously described [20].

EL4 (ATCC TIB-39) is a murine T lymphoma cell line. MICA-transfected EL4 (EL4-MICA) and control EL4 cells were grown in RPMI 1640 supplemented with 10% FCS, glutamine, and antibiotics as previously described [3].

NKL cells were grown in RPMI medium (Biosource International, Rockville, MD) containing 100 units/mL IL-2, 10% human serum (Valley Biomedical, Winchester VA), 1% L-glutamine, 1% Pen/Strep, 1% sodium pyruvate (Biosource) and 1% NEAA. All cells were cultured at 37°C in the presence of 5% CO_2_.

### Reagents, antibodies, and recombinant cytokines

cPLA2 inhibitor AACOCF3 was purchased from Calbiochem (La Jolla, CA). Arachidonic acid was obtained from Sigma (Saint Louis, MO). Anti-CD94, anti-CD16, anti-CD3, and anti-NKG2D mAbs (clone 1D11, IgG1) with isotype-matched control IgG1 were purchased from BD Pharmingen (San Diego, CA). PE-conjugated anti-NKG2D 1D11 and FITC-conjugated anti-CD107a were purchased from eBioscience (San Diego, CA). Anti-phospho-cPLA2 was purchased from Cell Signaling Technology (Beverly, MA). Anti-β-actin mAb was purchased from Sigma (St. Louis, MO). Human IL-15 and IL-2 were purchased from BD Pharmingen (San Diego, CA).

### siRNA and transfection

Synthesized oligonucleotide of cPLA2 siRNA and control siRNA were purchased from Santa Cruz Biotechnology (Santa Cruz, CA). Human NK clone cells were electroporated with an Amaxa nucleofector (Amaxa, Köln, Germany) using an Amaxa human NK cell nucleofector kit (VPA-1005, program U-001). Cells were cultured for 24 to 48 hours post-transfection before being used for experiments. For siRNA transfection, 4x10^6^ cells per cuvette were transfected with 20 µM cPLA2 or control siRNA. Transfection efficiency ranged from roughtly 30-40% 24 hours after electroporation.

### Cell signaling

To investigate cPLA2 phosphorylation, cells were first serum-starved for 30 hours. Where CF3 was used, cells were pre-incubated for 30 min at 37°C with 20 µM CF3 prior to stimulation. To crosslink immunoreceptors, cells were incubated for 4 min at 37°C with the indicated monoclonal antibody before adding F(ab’)_2_ GAM for the indicated duration at 37°C. Cells were lysed for 20 min in cold lysis buffer containing protease and phosphatase inhibitors (50 mM Tris-HCl at pH 7.5, 150 mM NaCl, 1% Triton-X100, 1 mM EDTA, 1 mM Na _3_VO_4_, 1 mM NaF, and protease inhibitor cocktail tablets). Cellular debris was removed by centrifugation at 15,000 RPM for 20 min at 4°C. Total lysates were subjected to SDS-PAGE electrophoresis and transferred to PVDF membranes (Biorad). Proteins were then detected by using the indicated antibodies followed by HRP-conjugated donkey anti-rabbit (HRP-DAR) Abs (Jackson Immunoresearch Laboratories) using the enhanced chemiluminescence (ECL) kit from Amersham Pharmacia Biotech. To examine perinuclear translocation of cPLA2 after stimulation, nuclear fractions were extracted according to the instructions of the NE-PER nuclear and cytoplasmic extraction kit (Pierce Biotechnology, Rockford, IL).

### Cytotoxicity assay

Standard 4h chromium-release assays were performed as previously described using P815 cells (a Fcg^+^ mouse mastocytoma, ATCC, Rockville, MD), EL4-MICA1, or control EL4 cells at the indicated effector/target ratios in duplicate wells. For Fc-dependent redirected cytotoxicity, effectors and targets were incubated in the presence of soluble anti-NKG2D and isotype IgG control antibodies at the indicated concentration. Chromium release was measured using a scintillation counter (Packard, Meriden, CT). Maximum release was determined by addition of detergent (10% SDS) and spontaneous release ranged from 5 to 10% of the maximum. The percentage of specific cytotoxicity was calculated using the formula 100x(CPM experimental-CPM spontaneous)/(CPM maximum-CPM spontaneous). Where indicated, effector cells were treated for 30 min prior to and during the assay with an inhibitor and/or arachidonic acid.

### Arachidonic acid release assay

1x10^6^/mL NKL or fresh NK cells separated from the joint fluid of PsA patients were labeled with 0.2 µCi ^3^H AA (specific activity 62.5 Ci/mmol, PerkinElmer Life Science, Boston, MA) in RPMI 1640 with 0.2% FCS at 37°C in 5% CO_2_ for 2 hours. After labeling, cells were washed three times to remove free ^3^H AA and, where indicated, pretreated with inhibitor for 30 min in medium with 0.2% bovine serum albumin (BSA). Labeled effector cells were stimulated with control EL4 cells or MICA-transfected EL4 cells (MICA/EL4) at an effector/target ratio of 2:1, 1:1, or 0.5:1 at 37°C. Supernatants and cell pellets were collected separately by centrifugation. ^3^H AA was measured with a scintillation counter. Percentage of ^3^H AA release was calculated as (supernatant^3^H/(supernatant ^3^H+ pellet ^3^H)) x100

### Measurement of granule release by BLT esterase assay

Granule release was evaluated as previously described. In brief, NKL cells were suspended in RPMI medium containing 2% FCS, then incubated in a 12 well plate mixed with EL4 and MICA/EL4 targets at an effector/target ratio of 1:1 at 37°C for 4 hours. Maximum granule release was determined using 1% Triton X-100. Supernatants were evaluated for esterase secretion using a standard N-benzyloxycarbony lysine thiobenzyl ester (BLT, Calbiochem, San Diego, CA). The percentage of BLT esterase activity was calculated using the following equation: 100x(experimental BLT esterase release-spontaneous BLT esterase release)/(maximum BLT esterase release-spontaneous BLT esterase release).

### Flow cytometric analysis

For surface staining, cells were incubated with fluorochrome-conjugated antibodies according to standard protocols. For intracellular staining, cells were fixed and permeabilized using a BD Biosciences kit (Palo Alto, CA). Fluorescence was analyzed on a six-color FACSCanto (BD) with quadrants set to score as negative >99% of control Ig-stained cells.

### Statistical analysis

Mixed effects models were constructed for all bar graphs in the figures. The treatment variable was included as a fixed predictor. Experimental plate was included as a random predictor in the model. The outcome variable, specific lysis, was log transformed after checking normality of the residuals from the mixed model. Tukey-adjusted pairwise comparisons are reported unless otherwise noted. The main effect of treatment was statistically significant for all conditions tested (p<0.001).

## Results

### cPLA2 is activated in NK cells and is critical for the production of AA and for cytolysis in response to NKG2D

**Figure 1:**
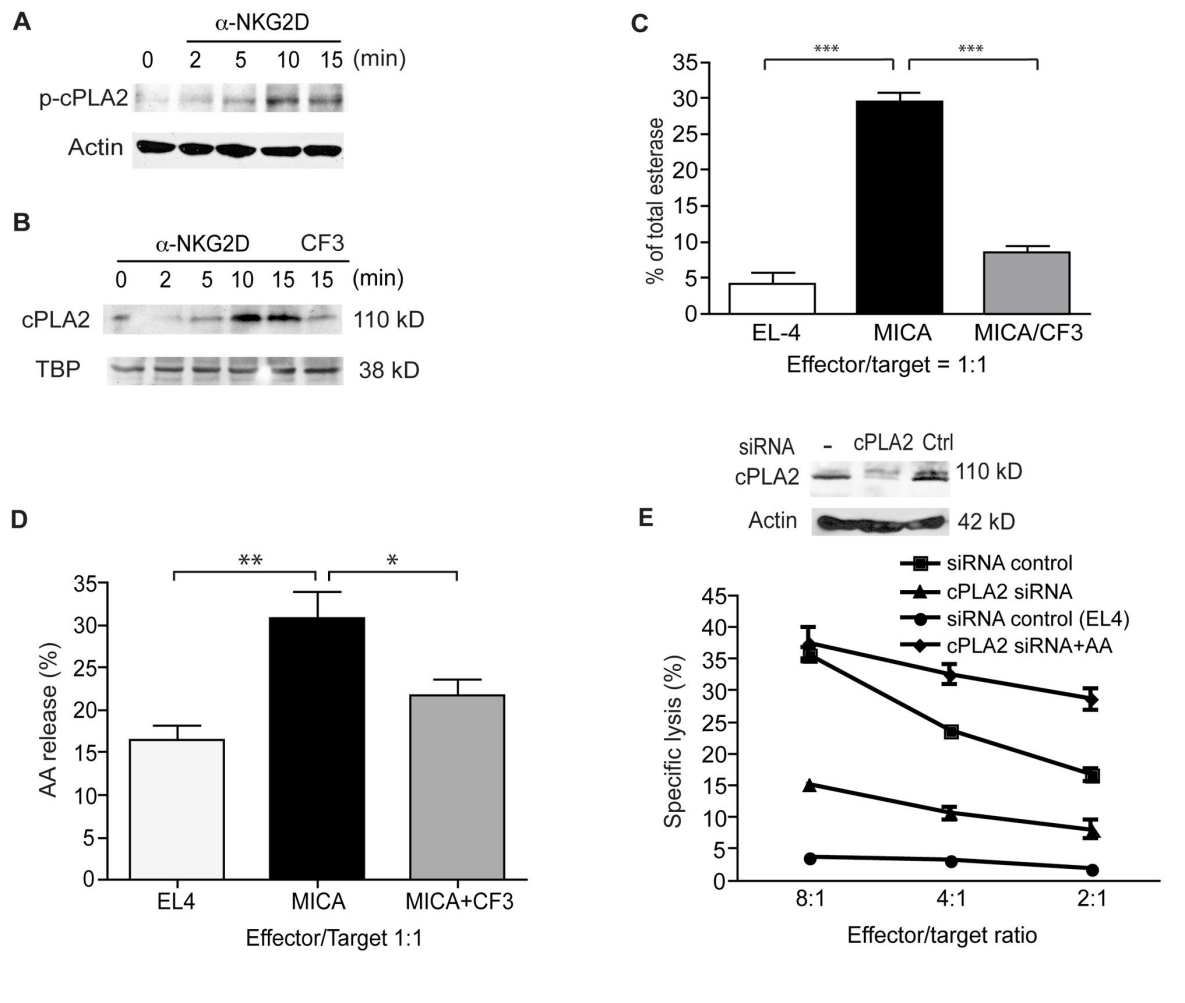
cPLA2 is activated in NK cells and is critical for the production of AA and for cytolysis in response to NKG2D. (**A**) cPLA2 is phosphorylated in response to NKG2D stimulation in NKL cells. Cells were stimulated with an anti-NKG2D mAb for the indicated time and probed for cPLA2 phosphorylation using a phospho-cPLA2-specific antibody. Data are representative of three independent experiments. (**B**) cPLA2 translocates to the nucleus in response to NKG2D stimulation in NKL cells. Nuclear extracts were probed using an anti-cPLA2 mAb. TATA binding protein (TBP) is shown as a loading control. Data are representative of three independent experiments. (**C**) NKG2D-mediated cytotoxicity of NKL cells was dramatically reduced upon inhibition of cPLA2 using the pharmacological inhibitor AACOCF3 (CF3). Data are means +/- SD of three independent experiments. (**D**) NK clones activated by NKG2D release arachidonic acid (AA) in a cPLA2-dependent manner. Data are means +/- SD of three independent experiments. (**E**) siRNA knockdown of cPLA2 abrogated NKG2D-mediated cytolysis. Scrambled control siRNA is shown as a control. Inset shows a representative western blot using a cPLA2 mAb, demonstrating efficient knockdown of cPLA2 after transfection with cPLA2 but not control siRNA. Transfected NKL cells were incubated with MICA-expressing or control EL4 target cells. Cytotoxicity data are means +/- SD of three independent experiments.

To assess a potential role for cPLA2 during NKG2D activation in NK cells, we first investigated its effects in the NKL cell line, which is derived from a large cell leukemia and is morphologically and functionally similar to an activated NK cell [21]. NKL cells were treated with 1 µL anti-NKG2D mAb and total lysates were analyzed for the presence of phosphorylated cPLA2 (p-cPLA2) (Figure 1A). We noted clear upregulation of p-cPLA2 after activation of NKG2D (Figure 1A). Furthermore, we performed nuclear protein extraction on NKL cells after NKG2D stimulation and upon assessment for cPLA2 found that translocation of cPLA2 was increased in response to NKG2D signaling (Figure 1B). Additionally, inhibition of cPLA2 using the pharmacological inhibitor CF3 blocked translocation to the nucleus (Figure 1B).

We then tested whether cPLA2 had an impact at a functional level. NKL cells exposed to target cells expressing MICA released esterase much more readily compared to those exposed to controls (Fig. 1C). We also generated NK clones from primary NK cells isolated from the blood of healthy controls, verifying that NKG2D crosslinking had the same effect on cPLA2 (Fig. S1A and data not shown). Using these clones (Fig. 1D) and NKL cells (Fig. S1B), we assessed whether NK cells would produce AA in response to NKG2D, noting that those exposed to targets expressing MICA produced significantly greater quantities. Significantly lower levels of esterase (Fig. 1C) and AA (Fig. 1D) were produced when cells were incubated with CF3, underscoring the importance of cPLA2. Finally, we tested whether cPLA2 has an impact on cytotoxicity in NK cells. Using cPLA2 siRNA, we were able to achieve efficient knockdown in our NK clones (Figure 1E inset) and found that cPLA2 ablation resulted in a marked decrease in MICA-expressing target cell killing (Figure 1E). The addition of exogenous AA resulted in a restoration or enhancement of target killing at all effector/target ratios tested, demonstrating that the importance of cPLA2 is tied to its ability to produce AA (Figure 1E). We obtained similar results using pharmacological inhibitors in NKL cells (Figure S1C).

Overall, these data demonstrate that cPLA2 plays a crucial role in NKG2D-mediated killing in NK clones and cell lines, mirroring our previous findings in CD8 effector T cells.

### Freshly isolated blood NK cells require an additional signal to lyse targets efficiently through NKG2D

**Figure 2:**
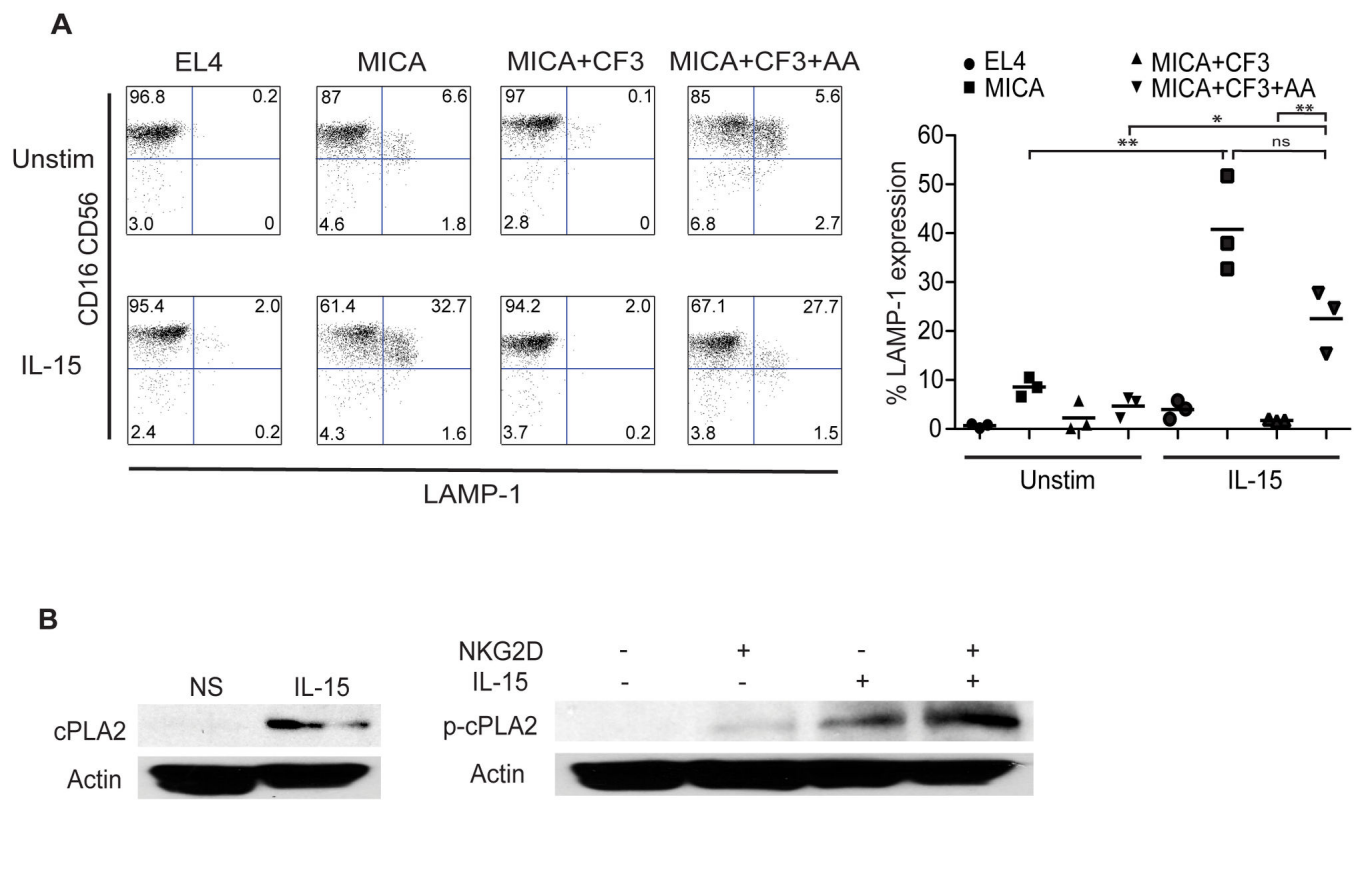
Freshly isolated blood NK cells require an additional signal to lyse targets efficiently through NKG2D. (**A**) Peripheral blood NK cells fail to kill via NKG2D in the absence of IL-15, but upon stimulation with 10 ng/mL IL-15 they are able to lyse targets in a cPLA2-dependent manner. Blood NK cells from healthy controls were isolated by negative selection and are gated on a CD3^-^CD94^+^ background. FACS plots are representative of three independent experiments, summarized on the right. (**B**) Blood NK cells upregulate cPLA2 basally and are primed to activate cPLA2 more robustly upon activation of NKG2D. Peripheral blood NK cells isolated from healthy controls were stimulated overnight with IL-15 and/or an anti-NKG2D mAb for four hours where indicated, then probed for cPLA2 (left) or phospho-cPLA2 (right). Data are representative of three independent experiments.

To test whether blood NK cells were fully licensed effector cells capable of killing through NKG2D, we assessed the ability of freshly isolated NK cells to degranulate in response to MICA-expressing target cells. We noted that these cells failed to degranulate at high levels (Figure 2A, top panels). However, when these cells were incubated with IL-15, they strongly degranulated in a cPLA2 dependent manner in response to MICA specifically (Figure 2A, bottom panels). We hypothesized that freshly isolated NK cells are unable to sufficiently activate cPLA2 to induce degranulation and that this defect is overcome upon IL-15 simulation. We first assessed the level of cPLA2 expression in NK cells isolated from the blood before and after stimulation. Freshly isolated blood NK cells were incubated with IL-15 for 48 hours and cPLA2 levels determined by western blot. Strikingly, the levels of cPLA2 expression were low in blood NK cells and reached significant levels only after IL-15 stimulation (Figure 2B, upper panel). Paralleling total cPLA2 expression, we found that cPLA2 was phosphorylated upon NKG2D stimulation at much higher levels after priming with IL-15, suggesting that NK cells from the blood require additional signals to induce high levels of cPLA2 expression and activation.

Overall, these data demonstrate that resting NK cells from the blood are not capable of killing stressed target cells through NKG2D alone. The presence of IL-15 is required for resting NK cells to become fully cytotoxic.

### NK cells from the joints of patients with psoriatic arthritis are aberrantly activated and are in the effector phase

**Figure 3:**
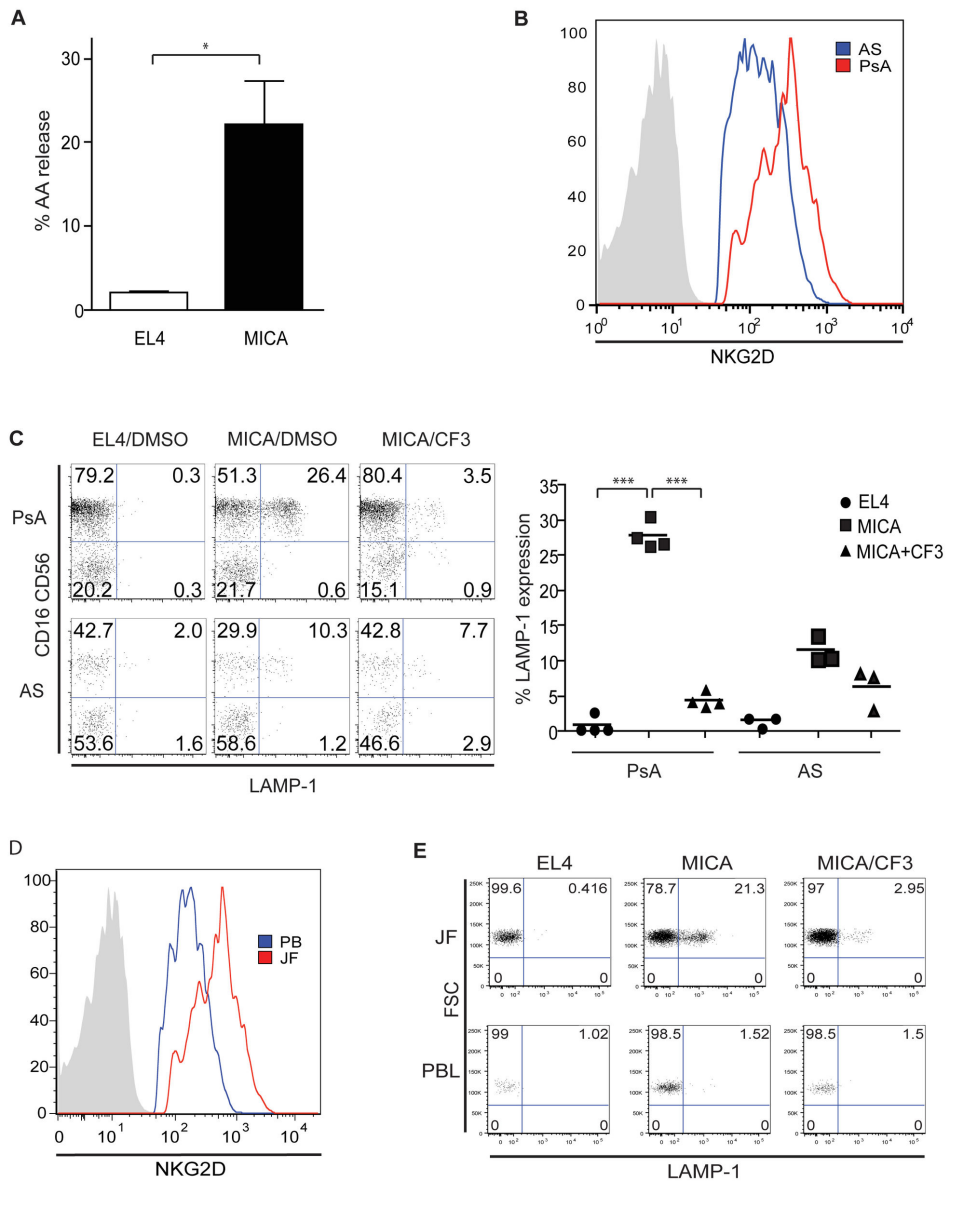
NK cells from the joints of psoriatic arthritis patients are aberrantly activated and are in the effector phase, and this process is dependent on cPLA2. (**A**) NK cells isolated from the joints of PsA patients are primed to be responsive to MicA, as measured by release of AA. Data are means +/- SD of three independent experiments. (**B**) NKG2D is strongly upregulated by NK cells isolated from the joints of PsA patients as compared to NK cells from the joints of patients with AS. An isotype control is shown in gray. Data are representative of three independent experiments. (**C**) Inhibition of cPLA2 significantly reduces the NKG2D-mediated cytotoxic potential of NK cells isolated from PsA but not AS patients. Cells are gated on a CD3^-^CD94^+^ background. FACS plots are representative of at least three independent experiments, shown in the right panel. (**D**) NKG2D expression is upregulated on NK cells from the joints but not those from the peripheral blood of PsA patients. An isotype control is shown in gray. Data are representative of three independent experiments. (**E**) Joint fluid but not peripheral blood NK cells from PsA patients degranulate in response to NKG2D in a cPLA2-dependent manner. Cells are gated on a CD3^-^CD16^+^CD56^+^CD94^+^ background. Data are representative of three independent experiments.

We then wanted to test whether NK cells from the joints of PsA patients were capable of killing via NKG2D. First, we assessed the ability of freshly isolated joint NK cells from PsA patients to produce AA, noting that they released significantly higher levels when exposed to MICA-expressing target cells compared to control targets (Figure 3A). We then wanted to see if this phenotype was specific to PsA patients or was characteristic of joint NK cells as a whole, using NK cells from the joints of patients with ankylosing spondylitis (AS) as a control. AS is an inflammatory disorder of the tendons, and patients have no irregularities in their synovial tissue, making it an ideal control disease. We found that cells from patients with PsA expressed higher levels of NKG2D as measured by flow cytometry (Figure 3B). To investigate the functional impact, we assessed the capacity of NK cells from PsA and AS patients to degranulate via NKG2D, noting that those from PsA patients had much higher levels of LAMP-1 after incubation with MICA-expressing targets, and that this was dependent on cPLA2 (Figure 3C).

We then questioned whether the NK cells of PsA patients were systemically altered or if those isolated from the joints were distinct. Flow cytometry revealed that NKG2D expression was considerably higher on NK cells isolated from the joints compared to those isolated from the blood of PsA patients, suggesting that the microenvironment of the inflamed joint was capable of driving upregulation of NKG2D (Figure 3D). To test whether this was also dependent on cPLA2, we incubated freshly isolated NK cells (gated as CD3^-^CD94^+^) from the joints and blood of PsA patients with CF3, noting that the NKG2D-mediated degranulation of joint NK cells was ablated upon inhibition of cPLA2 (Figure 3E).

Overall, these data suggest that NK cells from the joints of PsA patients are already in an effector phase, since they are capable of killing target cells expressing MICA in absence of a second signal. Furthermore, these data emphasize the importance of the joint microenvironment in influencing immune responses, since joint but not blood NK cells from PsA patients are primed to kill via NKG2D.

### PsA patients have higher levels of IL-15 in their synovial tissue, providing an environment wherein NK cells are primed to the effector stage

**Figure 4:**
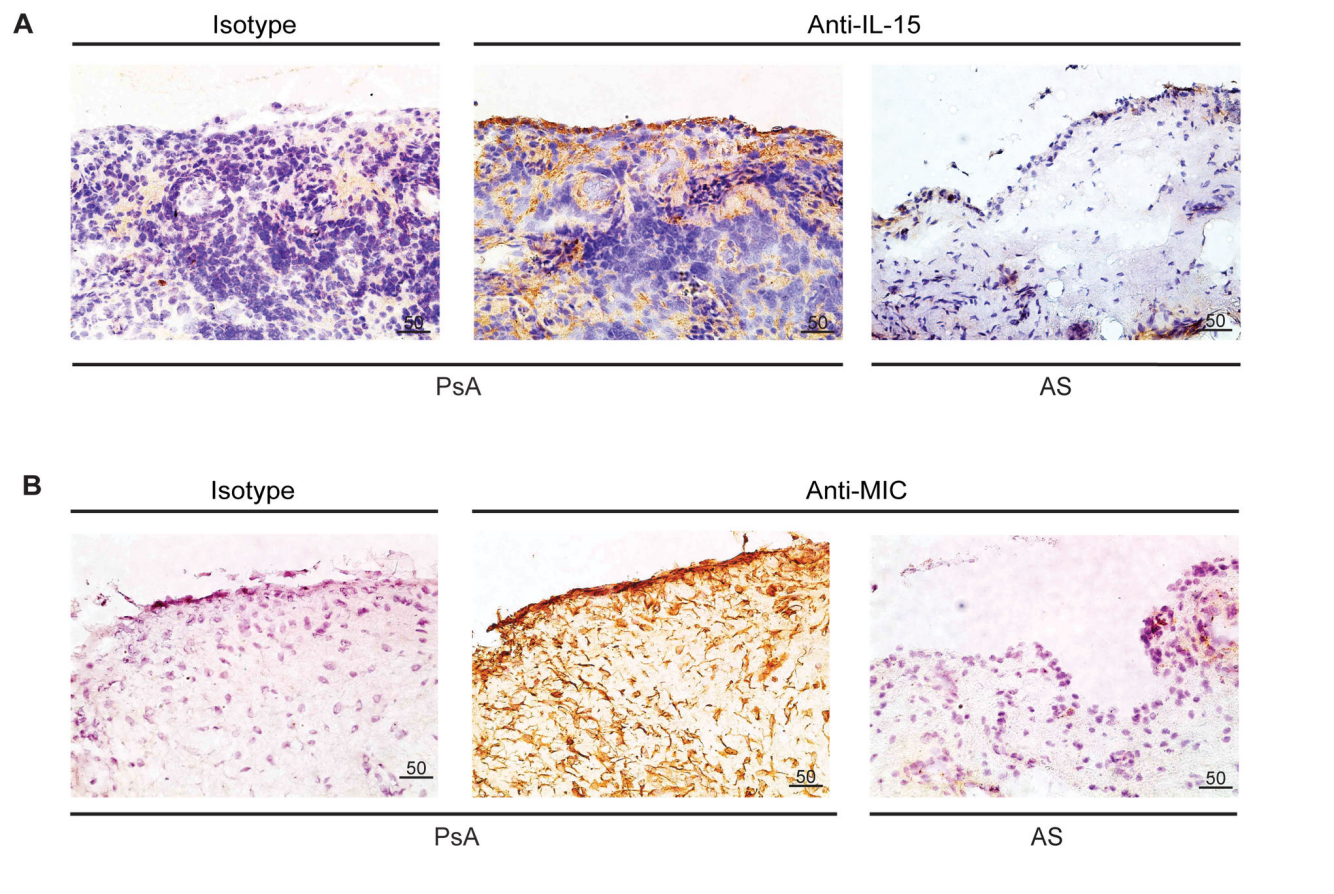
PsA patients have higher levels of IL-15 in their synovial tissue, providing an environment wherein NK cells are primed to the effector stage. (**A**) IL-15 is upregulated in the synovial tissue of patients with PsA but not AS, as shown by immunohistochemical staining using an anti-IL-15 mAb. Staining of a PsA tissue preparation with an isotype control antibody is shown on the left. Images are representative of at least three independent experiments. (**B**) Stress ligand levels are higher in the synovial tissue of PsA patients compared to patients with AS, as shown by immunohistochemical staining using an anti-MIC mAb. An isotype control is shown on the left as in (A). Images are representative of at least three independent experiments.

We then wanted to investigate potential explanations as to why PsA patient NK cells were more potently cytotoxic. Having previously found that IL-15 was capable of priming NK cells, we first assessed the levels of IL-15 in the synovial tissue of PsA patients, noting that there were much higher levels compared to AS patients (Figure 4A). We also found that PsA patient synovial tissue had significantly higher expression of MICA compared to AS patient tissue (Figure 4B). In conjunction with the increased IL-15 that is capable of priming NK cells to the effector phase, elevated MICA expression provides a potential explanation for joint tissue destruction and PsA pathogenesis.

## Discussion

Despite the fact that NK cells are widely considered to be prototypical effector cells, they require additional signals to fully unlock their cytolytic capabilities. Eric Long’s group demonstrated that the killing potential of resting NK cells is extremely limited until they are stimulated by additional exogenous signals, implying a multi-signal model of NK cell activation [22]. Activating signals include the release of ligands from inhibitory receptors, binding of ligands to activating receptors, and the activation of various cytokine receptors. In particular, IL-15 is an extremely potent activator of NK cells [23,24]. We have previously noted that localized tissue expression of MICA and IL-15 can lead to autoimmunity mediated by CD8^+^ effector T cells, likely due to the capability of IL-15 to drive the expression of NKG2D [4,6,25] and activate its cytolytic pathway [3]. Given these findings, we hypothesized that NK cells may be activated in the same manner, and that they may play a role in autoimmune pathogenesis, particularly in diseases that have been linked to IL-15. Furthermore, our study suggests that cPLA2 is critical for NKG2D-mediated cytolysis in NK cells, and that an important mechanism underpinning the ability of IL-15 to mediate cytolysis via NKG2D in NK cells is its ability to upregulate cPLA2 expression and activation.

The physiopathological relevance of these findings is supported by the observation that NKG2D can mediate cytolysis in NK cells isolated from the joints of PsA patients where IL-15 is upregulated. In accordance with a critical role for cPLA2 in freshly isolated IL-15-primed NK cells from synovial fluid, inhibition of cPLA2 was associated with a significant impairment of NKG2D-mediated cytolysis.

Our study also further addresses the question of whether PsA pathogenesis is innate or adaptive in nature. PsA is likely to be a heterogeneous disease, as the proportion of NK cells among synovial lymphocytes varies between 3 and 35% (data not shown and [18]), and it has been recently proposed that HLA heterogeneity plays a key role [13]. In particular, reports that PsA can develop in absence of autoreactive T cells [16] and is linked to IL-15 in mice [26] suggested that PsA can be driven by the innate rather than the adaptive immune system [17]. However, the mechanisms by which innate immunity and IL-15 could drive PsA have remained elusive. Our study indicates that IL-15 can drive joint inflammation and injury in PsA by arming the cytolytic NKG2D pathway in NK cells, causing them to either directly kill MIC expressing synovial cells or release AA, which has been tied to both the function and recruitment of granulocytes [27] and mast cells [28], two cell types thought to participate in the aberrant inflammatory responses of PsA [29]. However, these findings do not strictly exclude the considerable evidence for the presence of an adaptive immune response in PsA, which include class 1 HLA associations [13] and CD8 T clonal expansions [14,15]. Additionally, the role of NKG2D may not be restricted to NK cells, as we have evidence that NKG2D may mediate direct killing in IL-15-activated CD8^+^ T cell from PsA patients (Curran et al., manuscript in preparation) and we noted that PsA patient joint fluid lymphocytes were more potently cytotoxic than peripheral blood lymphocytes (Figure S2). Future studies will determine whether PsA may be subdivided into autoimmune PsA driven by expansion of CD8^+^ T cell clones [14,15] and innate immune PsA, in which NK cells have an active role in driving inflammatory pathogenesis.

Our study points to a potential role for NK cells in autoimmune disorders where specific tissue cell types would be destroyed based not on the recognition of self-antigens by T cells but instead on the expression of IL-15 and stress ligands, stimulating activating NK receptors such as NKG2D. By emphasizing the role of NK cells and importance of the tissue microenvironment with regard to influencing their activation, new and innovative therapies may be developed to treat PsA and other organ-specific autoimmune disorders in which IL-15 upregulation and NK cell infiltration are present in the target tissue.

## Supporting Information

Figure S1 **cPLA2 function is comparable between NKL cells and NK clones.** (**A**) cPLA2 is phosphorylated in response to NKG2D in NK clones. Total cPLA2 is shown as a loading control. Data are representative of three independent experiments. (**B**) NKL cells release AA in response to NKG2D only when cPLA2 is uninhibited. The left panel is representative of three independent experiments; the right panel shows means +/- SD of three independent experiments. (**C**) Inhibition of cPLA2 with CF3 significantly impaired anti-NKG2D mAb-redirected lysis of P815 targets by NKL cells. Addition of exogenous AA restored cytolysis. Data are representative of three independent experiments.
(TIF)

Figure S2 **Joint fluid but not peripheral blood cells lyse MICA-expressing target cells efficiently.** Lymphocytes from PsA patient joint fluid and blood were assessed for their ability to lyse MICA-expressing or control target cells. Addition of CF3 significantly impaired joint fluid cell cytolysis, while the addition of 100µM exogenous AA enabled peripheral blood cells to kill targets at comparable levels to joint fluid lymphocytes. Data are representative of three independent experiments.
(TIF)
