# Supplementary figures and images for: Interleukin 15 Primes Natural Killer Cells to Kill via NKG2D and cPLA2 and This Pathway Is Active in Psoriatic Arthritis
Source: PLoS One. 2013 Sep 25;8(9):e76292. doi: 10.1371/journal.pone.0076292 (PMC3783406; doi:10.1371/journal.pone.0076292)

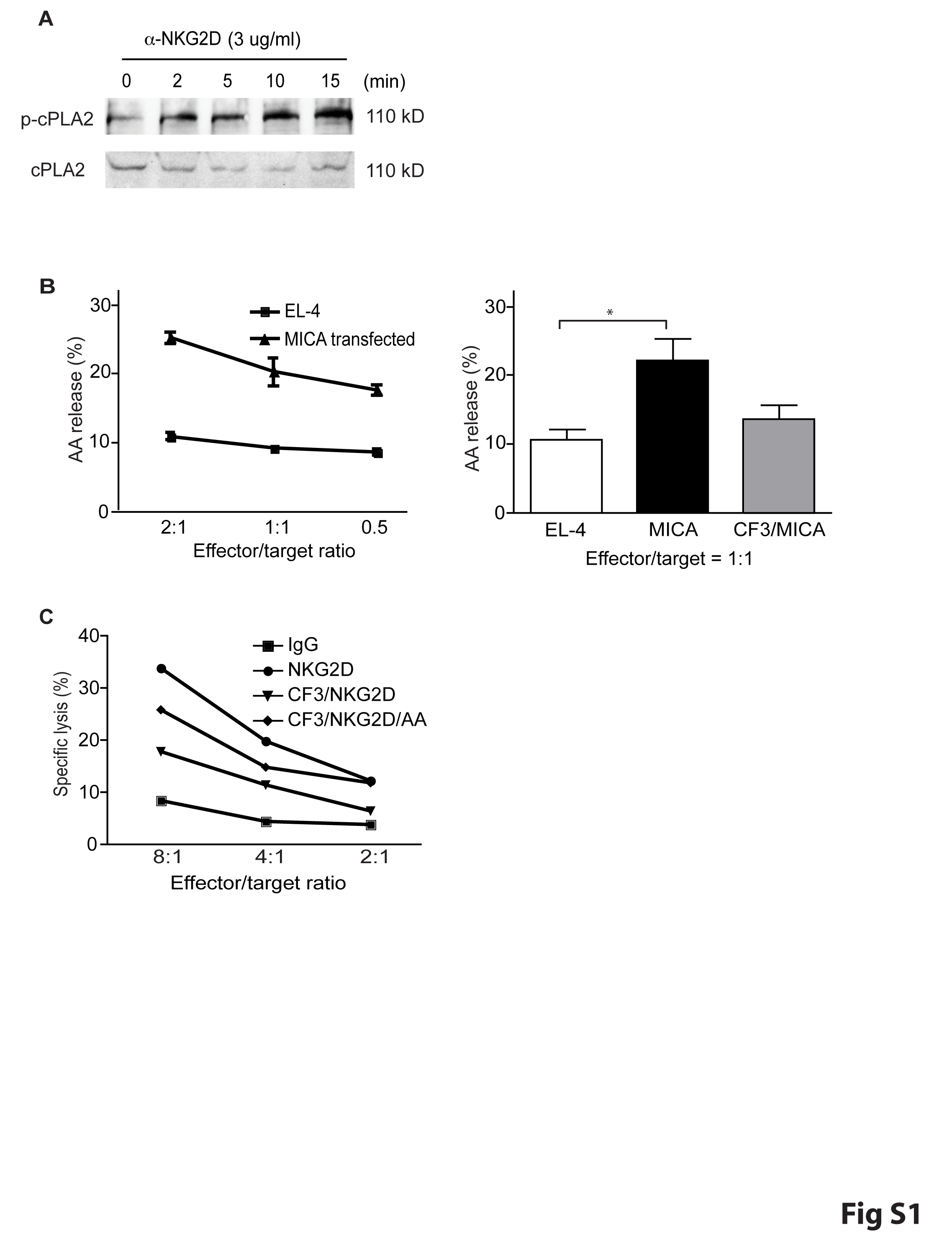

Supplement: Figure S1 — cPLA2 function is comparable between NKL cells and NK clones. (A) cPLA2 is phosphorylated in response to NKG2D in NK clones. Total cPLA2 is shown as a loading control. Data are representative of three independent experiments. (B) NKL cells release AA in response to NKG2D only when cPLA2 is uninhibited. The left panel is representative of three independent experiments; the right panel shows means +/- SD of three independent experiments. (C) Inhibition of cPLA2 with CF3 significantly impaired anti-NKG2D mAb-redirected lysis of P815 targets by NKL cells. Addition of exogenous AA restored cytolysis. Data are representative of three independent experiments. (TIF) [file pone.0076292.s001.tif]

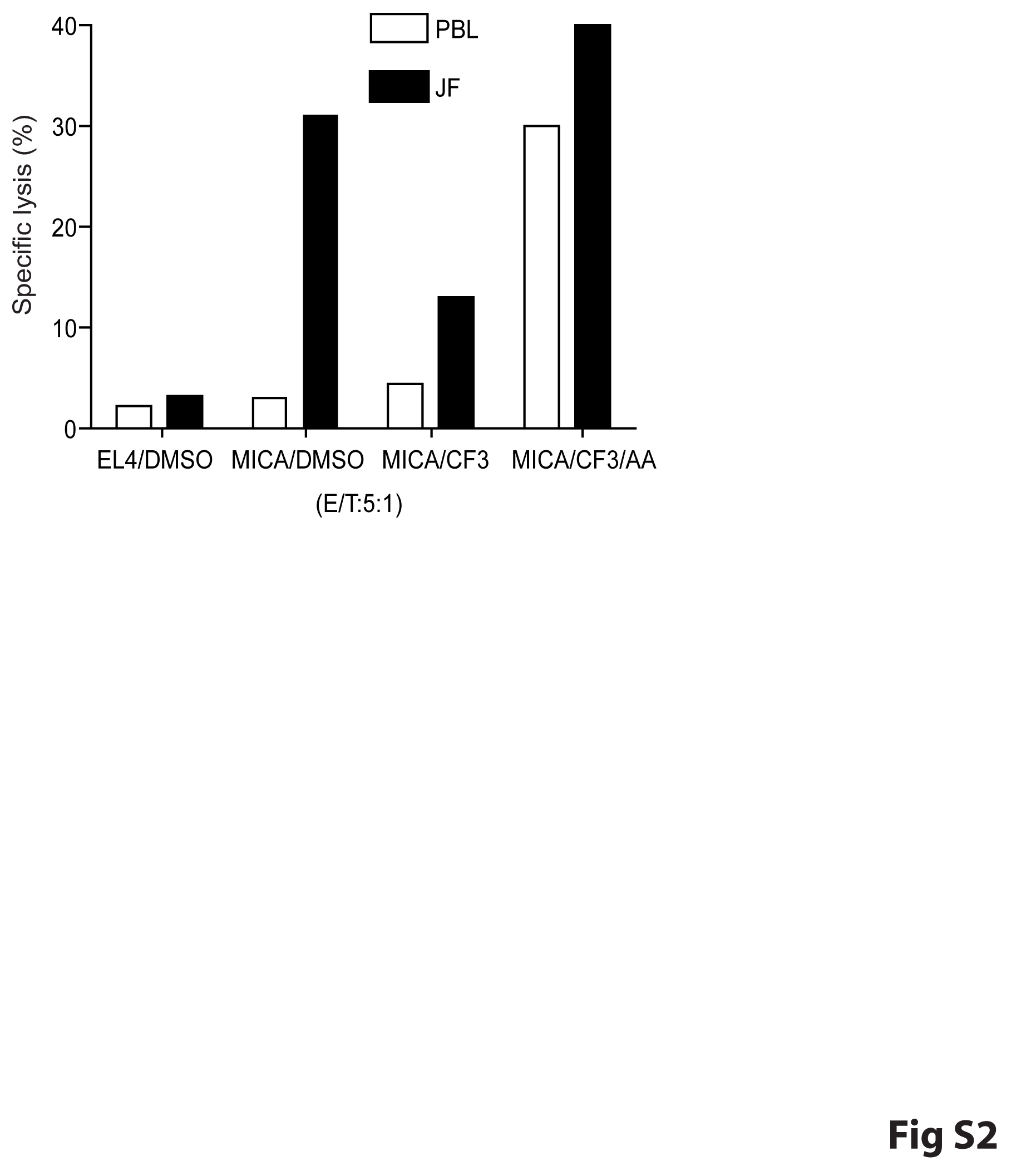

Supplement: Figure S2 — Joint fluid but not peripheral blood cells lyse MICA-expressing target cells efficiently. Lymphocytes from PsA patient joint fluid and blood were assessed for their ability to lyse MICA-expressing or control target cells. Addition of CF3 significantly impaired joint fluid cell cytolysis, while the addition of 100µM exogenous AA enabled peripheral blood cells to kill targets at comparable levels to joint fluid lymphocytes. Data are representative of three independent experiments. (TIF) [file pone.0076292.s002.tif]
